# Biomechanical analysis of load distribution in porcine hip joints at different acetabular coverages

**DOI:** 10.1186/s12891-024-07701-w

**Published:** 2024-07-24

**Authors:** Tetsuya Tachibana, Hiroki Katagiri, Junpei Matsuda, Nobutake Ozeki, Toshifumi Watanabe, Ichiro Sekiya, Tetsuya Jinno

**Affiliations:** 1 Department of Orthopedic Surgery, Dokkyo Medical University, Saitama Medical Center, 2-1-50 Minami-Koshigaya, Koshigaya City, Saitama 343-8555 Japan; 2 Department of Joint Surgery and Sports Medicine, Graduate School of Medical and Dental Sciences, Tokyo Medical and Dental University (TMDU), Tokyo, Japan; 3 Center for Stem Cell and Regenerative Medicine, Tokyo Medical and Dental University, Tokyo, Japan

**Keywords:** Biomechanics, Osteotomy, Hip dysplasia, Porcine, Femoroacetabular impingement

## Abstract

**Background:**

Developmental dysplasia of the hip causes secondary osteoarthritis. Finite element analysis suggests high hip joint contact pressure in patients with hip dysplasia and a reduction in contact pressure after periacetabular osteotomy. However, few biomechanical studies have examined the load distribution in the hip joint. This study aimed to investigate the biomechanical properties of load distribution in porcine hip joints at different acetabular coverages.

**Methods:**

Six porcine hip joints were analyzed using three models: 1) neutral coverage, 2) 15° under-coverage (defined as dysplasia model), and 3) 15° over-coverage created by varying the acetabular coverage. The load distribution was assessed using a pressure-mapping sensor system after applying a loading force of 100 N to the hip joint.

**Results:**

In the dysplasia model, the load was concentrated at the acetabular rim; in the neutral and over-coverage models, it was dispersed. The average contact pressure was significantly higher in the dysplasia model than in the neutral coverage model ([0.42 vs. 0.3 MPa]; *p* = 0.004). The contact area was significantly smaller in the dysplasia model than in the neutral coverage model ([250.7 vs. 345.0 mm^2^]; *p* = 0.004). No significant differences were observed in contact pressure or area between the neutral and over-coverage models.

**Conclusions:**

Insufficient acetabular coverage in the dysplasia model demonstrated higher contact pressure and smaller contact area than the neutral model. Conversely, the contact pressure and area in the over-coverage model did not differ significantly from those in the normal model. Therefore, surgeons should note that acetabular coverage overcorrection has limited effect; normalization is crucial during periacetabular osteotomy.

## Background

Developmental dysplasia of the hip (DDH) is the most frequent cause of secondary osteoarthritis (OA) [1]. The main contributing factors are mechanical stress concentration and instability. Estimates using finite element analysis and mathematical models have reported that patients with DDH have higher hip joint contact pressure at the acetabular rim than healthy patients [2]. Finite element analysis, however, has limitations due to its assumptions and simplifications that may not accurately reflect complex biological systems. Therefore, experimental studies are essential to validate and complement these theoretical models. In terms of instability, magnetic resonance imaging showed anterior subluxation of the femoral head in DDH [3]. However, there are few biomechanical studies on the load distribution in the hip joint.

Periacetabular osteotomy (PAO) is the standard treatment for correcting acetabular coverage and preventing OA in patients with DDH [4]. The effect of PAO on OA prevention is thought to be achieved by dispersing higher joint contact pressure by increasing the acetabular coverage [5, 6]. However, how hip joint pressure changes with increasing acetabular coverage remains unclear. While human cadaver experiments are ideal for studying load distribution in hip joints, limited resources make animal models a necessary preliminary step.

Therefore, this study aimed to establish a porcine model to investigate the biomechanical properties of the load distribution of the normal hip joint, and to create a dysplastic hip model and an over-coverage model to measure the load distribution of the joint. We hypothesized that the joint contact pressure in the dysplastic hip model would be higher, and the contact area would be smaller than in the normal model.

## Methods

### Materials

Animal experiments were performed in our institution's biomechanics laboratory in accordance with the regulations of the Institution’s Animal Care and Use Committee. Ethical approval by the committee was waived due to the ex vivo nature of this study. Six freshly frozen porcine hip joints from approximately 6-month-old commercially slaughtered pigs (strain and gender unknown; Tokyo Shibaura Zouki, Tokyo, Japan) were used in the experiments. Joints with cartilage or labral injuries were excluded. The specimens were procured to include the pubic symphysis and sacrum; they were stored at –20 °C and thawed at room temperature for 24 h before testing. Each specimen was dissected by an orthopedic surgeon with more than 10 years of experience in orthopedic surgery to preserve the hip joint capsule and its attachments to the pelvis and femur.

### Experimental setup

**Fig. 1:**
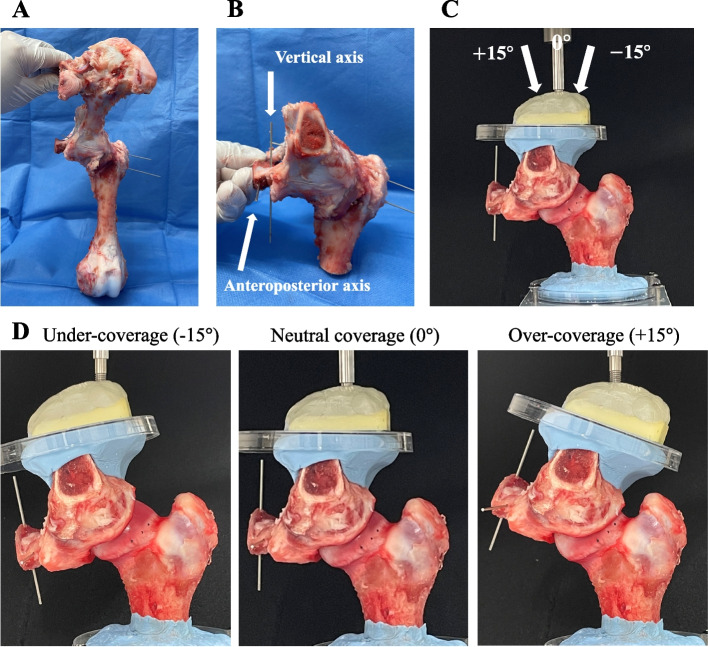
Experimental settings. **A** Anterior view of a left porcine hip joint. The sacrum and pubic symphysis were aligned in the sagittal plane (vertically). The pelvis and femur were fixed. **B** Anterior view of the left hip joint after cutting. Two Kirschner wires were inserted along the vertical and anteroposterior axis. **C** The left hip was fixed using an angle-changing device set at 0°. **D** The left hips were set at -15° (under-coverage model defined as dysplasia model), 0° (neutral coverage model), and + 15° (over-coverage model)

After the removal of the muscles from around the hip joint, the initial pelvic position was set by obtaining pelvic neutrality. The neutral position was maintained throughout the test, based on the method proposed by Fagotti et al. [7] (Fig. 1). The pelvis was placed on a table with the tip of the sacrum and the pubic symphysis aligned in the sagittal plane. A consistent neutral pelvic tilt was established by flexing or extending the pelvis until the sacral plate was vertically aligned (Fig. 1-A). The neutral rotation of the pelvis was visually confirmed by ensuring that there was no tension in the hip capsule [8].

Neutral rotation of the femur was obtained such that the axis connecting the center of the femoral head and the center of the femoral shaft coincided with the horizontal axis of the pelvis, as viewed from an upper standpoint. The hip was secured in this position using two Kirschner wires passing through the femur and pelvis.

After fixation of the femur to the pelvis, it was cut 8 cm distal to the tip of the greater trochanter and perpendicular to the femoral shaft. The anterior edge of the pelvic cutline was set 2 cm anterior to the anterior inferior iliac spine, the posterior edge was set at the base of the inferior pubic ramus, and the upper edge was set 3 cm above the joint space. Before cutting, a Kirschner wire was inserted into the pubic bone parallel to the vertical, horizontal, and anteroposterior axes of the pelvis to preserve these axes (Fig. 1-B). To ensure standardized hip alignment, the proximal femur was first fixed with polymethyl methacrylate and then secured to the platform of a custom universal testing machine. This testing machine system was set as described in a previous biomechanical study [9, 10]. With the femur fixed in the proper orientation, the pelvis was secured with polymethyl methacrylate and was secured to the testing machine. The hip capsule and the wires securing the orientation of the hip joint were removed after setting. The labrum and ligamentum teres were preserved (Fig. 1-C). A schematic representation of this procedure is shown in Fig. 1-D. For each setting, the specimen was compressed to 100 N and held in place [11]. A force of 100 N was selected for compressive loading. During pilot testing, forces greater than 100 N caused joint pressures that greatly exceeded the sensor’s upper measurement limits (2.34 MPa) in wide areas. Biomechanical testing was run in each of the three testing states: native coverage, defined as the neutral coverage model; 15 ^∘^ under-coverage, defined as the dysplasia model; and 15 ^∘^ over-coverage, defined as the over-coverage model. We changed the coverage by moving the pelvis along the femoral head while preventing pelvic rotation and tilting. For all specimens, the neutral (native coverage) state was tested first.

### Contact area and force measurement

A Tekscan pressure-mapping sensor 5027 system (Tekscan Inc. South Boston, MA, USA) was used to evaluate the distribution of the bearing force of the femoral head, which enabled electronic scanning of real-time force measurements. The sensors were calibrated and equilibrated using the Tekscan pressure calibration unit and correction software (I-SCAN, version 7.51-12I) according to the manufacturer's instructions. A new sensor was used for each specimen to ensure accuracy. The sensor was selected and placed to maximize the contact area in the hip joint and minimize wrinkling. The inner edge of the sensor placement was positioned just outside the attachment of the ligamentum teres, and the anterior edge of the sensor was aligned with the anterior edge of the contact area in the neutral position. To prevent movement during testing, the sensors were sutured to and stabilized in the soft tissue surrounding the femoral head. The load distribution was recorded during the application of the loading force. The wrinkling of the film over time, owing to the dry environment, was minimized using saline mist. The force measurements included maximum load, average contact pressure, and contact area. To ensure measurement reliability, we conducted test–retest experiments using two additional porcine hip joints in the neutral position, with 10 repeated measurements of contact pressure and contact area for each joint. The intraclass correlation coefficients for contact pressure and contact area were 0.91 and 0.96, respectively. All data was analyzed using MATLAB® (MathWorks, MA, USA).

### Statistical analysis

Statistical analyses were performed using the STATA 16 software package (StataCorp LP, College Station, TX, USA). All data are presented as means ± standard deviations. The contact pressure and area were compared using analysis of variance with Bonferroni correction for multiple comparisons. The level of significance was set at *p* < 0.05.

## Results

**Fig. 2:**
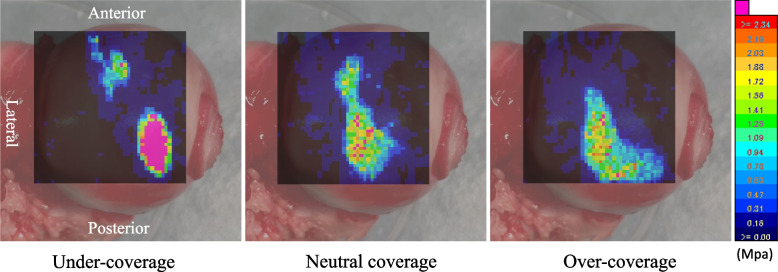
Load distribution changes in the three models. The load was concentrated on the acetabular rim in the under-coverage model. The load was dispersed, and the loading area was moved laterally in the neutral and over-coverage models

According to the representative images for each coverage model (Fig. 2), the load was concentrated in the area close to the acetabular rim in contact with the femoral head in the dysplastic model. In contrast, in the neutral and over-coverage models, the load was dispersed, and the loading area moved laterally.

**Fig. 3:**
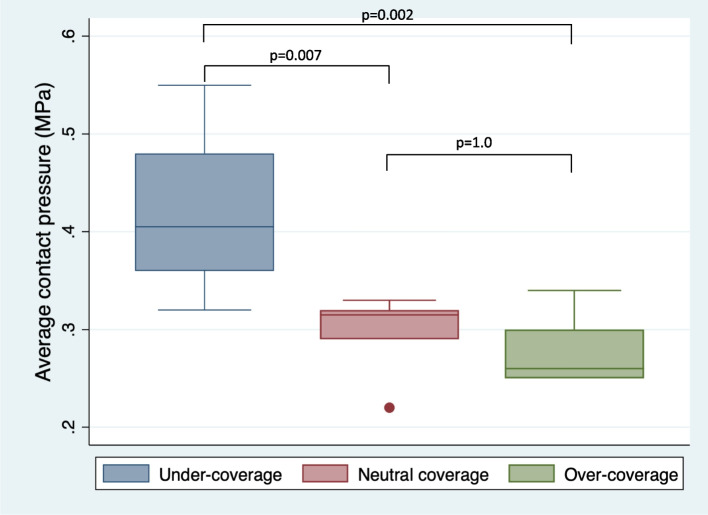
Average contact pressures in the porcine hip joint for the three models

The average contact pressure was significantly higher in the dysplasia model than in the neutral coverage model (0.42 ± 0.08 vs. 0.3 ± 0.04 MPa; *p* = 0.007) (Fig. 3). The average contact pressure was also significantly higher in the dysplasia model than in the over-coverage model (0.42 ± 0.08 vs. 0.28 ± 0.04 MPa; *p* = 0.002). However, no significant difference was observed in the average contact pressure between the neutral and over-coverage models (0.3 ± 0.04 vs. 0.28 ± 0.04 MPa; *p* = 1.0).

**Fig. 4:**
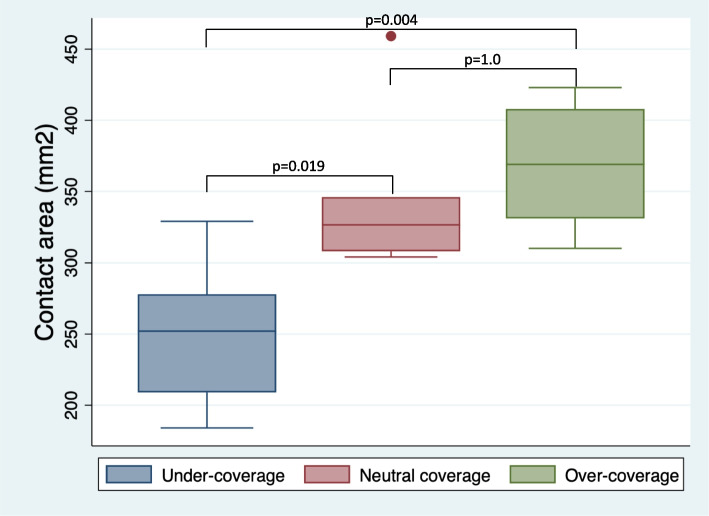
Contact area in the porcine hip joint for the three models

The contact area was significantly smaller in the dysplasia model than in the neutral coverage model (250.7 ± 52 vs. 345.0 ± 58 mm^2^; *p* = 0.019) (Fig. 4). The contact area was also significantly smaller in the dysplasia model than in the over-coverage model (250.7 ± 52 vs. 368.3 ± 43 mm^2^; *p* = 0.004). Conversely, no significant difference was observed in the contact area between the neutral and over-coverage models (345.0 ± 58 vs. 368.3 ± 43 mm^2^; *p* = 1.0).

## Discussion

In this study, we investigated the load distribution in porcine hip joints with different acetabular coverages using dysplasia, neutral, and over-coverage models. The most important finding was that the average contact pressure in the dysplasia model was significantly higher than that in the neutral model, and the contact area in the dysplasia model was significantly smaller than that in the normal model. These biomechanical evaluation results are consistent with our hypothesis. However, the contact pressure and area in the over-coverage model did not differ significantly from those in the neutral-coverage model.

Henak et al. reported the contact pressure in normal hip joints using cadaveric specimens [12], and our study's mapping of the contact area of the porcine hip joints was similar to that in their report. Furthermore, Henak et al. reported that the contact pressure in DDH is concentrated at the junction between the acetabular rim and labrum based on the finite element method [2]. Our contact pressure distribution results for the dysplasia model were also concentrated at the acetabular rim.

As insufficient coverage after PAO is thought to be a risk factor for conversion to total hip arthroplasty, surgeons tend to accept excessive correction of PAO [6, 13]. However, the results of this study indicate that over-coverage of the acetabulum has a limited effect on contact pressure reduction in the hip joint. Consistent with our results, Abraham et al. used a finite element model and reported that an increase in acetabular coverage did not necessarily yield a proportional reduction in contact pressure [14]. They suggested that surgeons should not assume a linear relationship between an increase in acetabular coverage and a reduction in contact pressure. Therefore, surgeons should consider that the effect of acetabular coverage overcorrection is limited when performing PAO.

The present study had a few limitations. First, this was an in vitro bench test using a porcine model rather than a human model. Porcine models are similar to humans in terms of cartilage thickness and joint-loading biomechanics [15]. However, our results may not be applicable in clinical practice. Second, our dysplasia model was created using normal porcine hips. Nepple et al. reported that three-dimensional acetabular morphology in DDH was different from that in normal hips, and several patterns of acetabular deficiency were common in adult patients with DDH [16]. Furthermore, the neutral alignment of the porcine hip in this study was based on human hip alignment protocols. Kitamura et al. reported that alignment in the sagittal plane could significantly influence joint contact pressure based on a finite element analysis [17]. However, differences in normal values of sagittal alignment between porcine and human hips may limit the transferability of our findings. Further biomechanical experiments involving human hips with DDH should be conducted. Third, the number of samples was small owing to limited specimen availability. Fourth, the study investigated acetabular coverage at -15°, 0°, and 15° varus-valgus alignments. The relatively large angle increments may not fully capture the sensitivity of contact area and pressure to acetabular coverage variations. Fifth, our study used average contact pressure and contact area to assess changes in the overall biomechanical environment within the hip joint, but data on maximum contact pressure were not obtained. Sixth, our system applied compressive load to the hip in a constrained manner. While we ensured that joint incongruity was avoided when varying acetabular coverage was applied, this constrained system might exhibit a pressure distribution in the joint that differs from the normal hip joint environment. Additionally, in the human hip, under loading conditions, the stabilizing effect of the hip abductors results in pressure being applied parallel to the femoral neck rather than purely vertically [18]. Therefore, the joint pressure distribution we demonstrated may differ from that observed under normal conditions. Seventh, the applied force in our study was 100 N, which is less than the load experienced during basic activity in the human hip. However, Li et al. reported that the pressure distribution pattern in the porcine hip remained stable for loading ≥ 100 N [11], indicating the relevance of our study's results despite the lower force applied. Future studies should aim to develop experimental systems capable of applying larger loads to investigate biomechanical responses further.

## Conclusions

Insufficient acetabular coverage in the dysplasia model demonstrated higher contact pressure and smaller contact area than in the normal model. In contrast, the contact pressure and area in the over-coverage model did not differ significantly from those in the normal model. Surgeons should consider that overcorrecting acetabular coverage has limited effectiveness, emphasizing the importance of normalizing acetabular coverage during PAO.

## Data Availability

All data generated or analysed during this study are included in this published article.

## Abbreviations

PAO: Periacetabular osteotomy
OA: Osteoarthritis
DDH: Developmental dysplasia of the hip
